# Clinical characteristics and prognostic risk factors of healthcare-associated pneumonia in a Korean tertiary teaching hospital

**DOI:** 10.1097/MD.0000000000008243

**Published:** 2017-10-20

**Authors:** June H. Ahn, Kwan H. Lee, Jin H. Chung, Kyeong-Cheol Shin, Choong K. Lee, Hyun Jung Kim, Eun Young Choi

**Affiliations:** a Department of Internal Medicine, Yeungnam University Medical Center, College of Medicine, Yeungnam University; b Division of Pulmonary and Critical Care Medicine, Department of Internal Medicine, Kyungpook National University Hospital, Kyungpook National University School of medicine, Daegu, South Korea.

**Keywords:** healthcare-associated pneumonia, mortality, pneumonia severity index

## Abstract

The 2016 American Thoracic Society and Infectious Diseases Society of America (ATS/IDSA) guidelines removed the concept of healthcare-associated pneumonia (HCAP). We examined whether the 2016 ATS/IDSA guidelines are applicable in Korea.

We conducted a retrospective, observational study of pneumonia patients who were hospitalized at a tertiary teaching hospital from March 2012 to February 2014. Identified pathogens that were not susceptible to β-lactams, macrolides, and fluoroquinolones were defined as community-acquired pneumonia drug-resistant pathogens (CAP-DRPs). We analyzed the risk factors for 28-day mortality and the occurrence rate of CAP-DRPs.

Of the 1046 patients, 399 were classified with HCAP and 647 with CAP. HCAP patients were older and had more comorbidities than CAP patients. Initial pneumonia severity index (PSI) was higher in patients with HCAP than with CAP. HCAP was associated with not only an increased rate of CAP-DRPs (HCAP, 19.8%; CAP, 4.0%; *P* < .001) but also an increased rate of inappropriate initial antibiotic therapy (IIAT) (HCAP, 16.8%; CAP, 4.6%; *P* < .001). HCAP was also associated with an increased 28-day mortality rate compared with CAP (HCAP, 14.5%; CAP, 6.3%; *P* < .001). In a multivariable analysis, PSI was an independent risk factor for 28-day mortality in HCAP patients (odds ratio 1.02, 95% confidence interval 1.01–1.04). CAP-DRPs and IIAT were not associated with mortality.

Patients with HCAP revealed higher rates of CAP-DRPs, IIAT, and mortality than patients with CAP. However, CAP-DRPs and IIAT were not associated with mortality. PSI was the main predictive factor for 28-day mortality in patients with HCAP.

## 1 Introduction

The 2005 American Thoracic Society and Infectious Diseases Society of America (ATS/IDSA) guidelines first introduced the concept of healthcare-associated pneumonia (HCAP). According to these guidelines, elderly patients with HCAP who have frequent contact with the healthcare system are at high risk of multidrug-resistant (MDR) pathogens. Dual antipseudomonal antibiotics plus anti-methicillin-resistant *Staphylococcus aureus* (MRSA) antibiotics were recommended regardless of the severity of the illness in these patients.^[1]^

Although these guidelines have been useful for treating pneumonia for a decade, there are some challenges to this concept of HCAP. Several studies have demonstrated that the HCAP definition did not predict the occurrence of MDR pathogens.^[2–4]^ Furthermore, although patients with HCAP are potentially at risk for MDR pathogens, underlying individual patient characteristics are also important risk factors.^[5]^ Based on these findings, the 2016 ATS/IDSA guidelines removed the concept of HCAP.^[6]^

However, HCAP itself is heterogeneous and the utility of the HCAP concepts varies according to geographical region, where countries differ in terms of patient characteristics and healthcare systems. Therefore, it is uncertain whether the new ATS/IDSA guidelines are applicable in Korea. To test this, we examined whether removal of the concept of HCAP from the 2016 ATS/IDSA guidelines was appropriate for Korea.

This study evaluated the baseline characteristics, pathogen distribution, antibiotic therapy, and clinical outcomes of HCAP and community-acquired pneumonia (CAP) patients. We also clarified the risk factors for MDR pathogens in pneumonia patients at a tertiary teaching hospital in South Korea.

## 2 Methods

### 2.1 Study design and subjects

We conducted a retrospective observational study of 1046 patients with pneumonia who were hospitalized at Yeungnam University Hospital (a 930-bed, university-affiliated, tertiary referral hospital in Daegu, South Korea), between March 2012 and February 2014.

Over the study period, all consecutive patients with pneumonia admitted to the hospital via the emergency or outpatient department were eligible. This study excluded patients with hospital-acquired pneumonia (HAP) that developed after being hospitalized for more than 48 hours, those less than 18 years, immunocompromised patients (such as those with neutropenia after chemotherapy or human immunodeficiency virus infection), and patients with *Mycobacteria tuberculosis* infection.

This study was conducted in accordance with the declaration of Helsinki. As this study is retrospective, no ethical committee approval was required for its conduction, which is in compliance with the institutional and national policies concerning research approvals. No informed consent was obtained because this study involved a chart review, and patient records were anonymized before analysis.

### 2.2 Definitions and outcome variables

Pneumonia was defined as the presence of new radiographic infiltrate and at least 2 of the following criteria: fever (>38 °C) or hypothermia (≤35 °C), new cough with or without sputum production, pleuritic chest pain, dyspnea, and altered breath sounds on auscultation.^[7]^ HCAP and CAP were defined according to the 2005 ATS/IDSA guidelines.^[1,8]^

HCAP included patients with any of the following conditions: hospitalization in an acute care hospital for ≥2 days within 90 days of the infection; residence in a nursing home or long-term care facility; infusion therapy, such as intravenous antibiotic therapy, chemotherapy, or wound care within 30 days of a current infection; or chronic dialysis, including hemodialysis and peritoneal dialysis within 30 days of a current infection. CAP included any patients with pneumonia who did not meet any of the criteria for HCAP.^[1]^ Community-onset pneumonia was defined as pneumonia in the community and up to 48 hours into hospital admission, and included both HCAP and CAP.^[9]^

In this study, identified pathogens that were not susceptible to β-lactams, macrolides, and fluoroquinolones were defined as community-acquired pneumonia drug-resistant pathogens (CAP-DRPs), as previously reported.^[5]^ Inappropriate initial antibiotic therapy (IIAT) was defined if the identified pathogens were not susceptible to the initially prescribed empirical antibiotics, based on an in vitro antibiotic susceptibility test. The severity of pneumonia in each group was determined using the pneumonia severity index (PSI) scores on hospital day 1.

Patients were classified into HCAP and CAP. We compared clinical characteristics, pneumonia severity, pathogen distribution, antibiotic regimens, clinical outcomes such as 28-day mortality, intensive care unit (ICU) admission, need for mechanical ventilator, hospital stay, and frequency of IIAT between these 2 groups. We identified independent risk factors for 28-day mortality of pneumonia patients. We also identified independent risk factors for drug-resistant pathogens of pneumonia patients.

### 2.3 Microbiological studies

Microbiological studies were performed using respiratory specimens and blood cultures using standard microbiological procedures. Gram staining and cultures were performed using sputum, tracheal aspirate, and bronchial washing fluids that were obtained by bronchoscopy. Respiratory specimens were cultured in a semi-quantitative manner, and an etiological diagnosis was established when a predominant microorganism was isolated from group 4 or 5 sputum, according to Murray and Washington's grading system. A pathogen was considered causative based on blood culture results if the same microorganism was also identified in a respiratory specimen or if no other source could be identified for the positive blood culture. In addition, a positive pneumococcal antigen (Alere BinaxNOW *S.pneumonia* Test; Binax Inc., Scarborough, ME) and a positive antibody titer (immunoglobulin M) for *Mycoplasma* were considered to indicate etiological pathogens. The antibiotic sensitivity of all isolates was determined using an automated bacterial identification and antibiotic susceptibility testing (VITEK2; bioMerieux Inc., Lyon, France). Microbiologic test results were independently reviewed by two investigators (JHA and EYC) using the electronic medical records.

### 2.4 Antibiotic therapy

Antibiotic therapy was initiated according to the 2005 ATS/IDSA guidelines,^[1]^ but the detailed antibiotic therapy was initiated according to the attending physician's decision only, taking into consideration the severity of the disease and underlying conditions. When a pathogen was identified, antibiotic therapy was modulated according to the susceptibility test results. The appropriateness of antibiotic therapy was analyzed according to the susceptibility test criteria. Antibiotic therapy was initiated after at least blood culture, sputum culture samples were done, and all the patients had microbiologic results available.

### 2.5 Statistical analysis

The *χ*^*2*^ test or Fisher exact test was used to compare categorical variables. Continuous variables were analyzed using Student *t* test or the Mann–Whitney *U* test. Multivariable logistic regression analyses were performed to identify independent risk factors associated with 28-day mortality in pneumonia patients and to identify risk factors for the occurrence of CAP-DRPs, as measured by the odds ratio (OR) with 95% confidence intervals (CIs). In all analyses, *P* < .05 was considered to indicate significance by 2-tailed tests. All statistical procedures were performed using SPSS software (version 18.0; SPSS Inc., Chicago, IL).

## 3 Results

### 3.1 Baseline characteristics

During the study period, 1046 patients who required admission care for pneumonia were eligible for the study: 399 with HCAP (38.1%) and 647 with CAP (61.9%). The criteria for the HCAP group are shown in Table 1. The most common HCAP criterion met was the administration of antibiotics within the past 30 days of the infection (90.5%). The demographic and baseline clinical characteristics of the patients with HCAP and CAP are presented in Table 2. Males predominated in both groups (70.7% in HCAP vs 63.8% in CAP, *P* = .02). Patients with HCAP were older than those with CAP (71.7 ± 12.0 vs 67.7 ± 15.2 years, *P* < .001). HCAP patients were significantly more likely to have comorbidities, such as congestive heart failure (11.0% vs 5.6%, *P* = .001), cerebrovascular disease (31.3% vs 12.4%, *P* < .001), and dementia (12.3% vs 4.9%, *P* < .001), than CAP patients. However, chronic lung disease (24.1% vs 35.1%, *P* < .001) occurred more frequently in patients with CAP. The frequencies of previous use of antibiotics, use of gastric acid-suppressive agents, tube feeding (nasogastric tube and gastrostomy tube), nonambulatory status (wheelchair for ambulation and being bedridden), and drowsiness or stupor were higher in patients with HCAP than in those with CAP. PSI was significantly higher in patients with HCAP than in those with CAP (113.5 ± 29.6 vs 97.4 ± 29.5, *P* < .001).

**Table 1:**
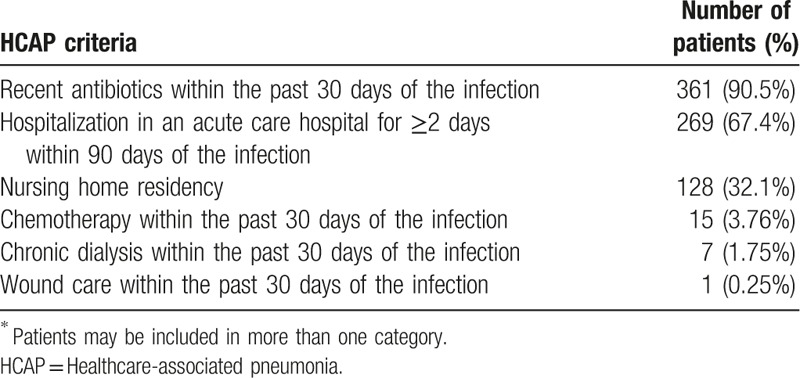
Data of 399 patients with HCAP^∗^.

**Table 2:**
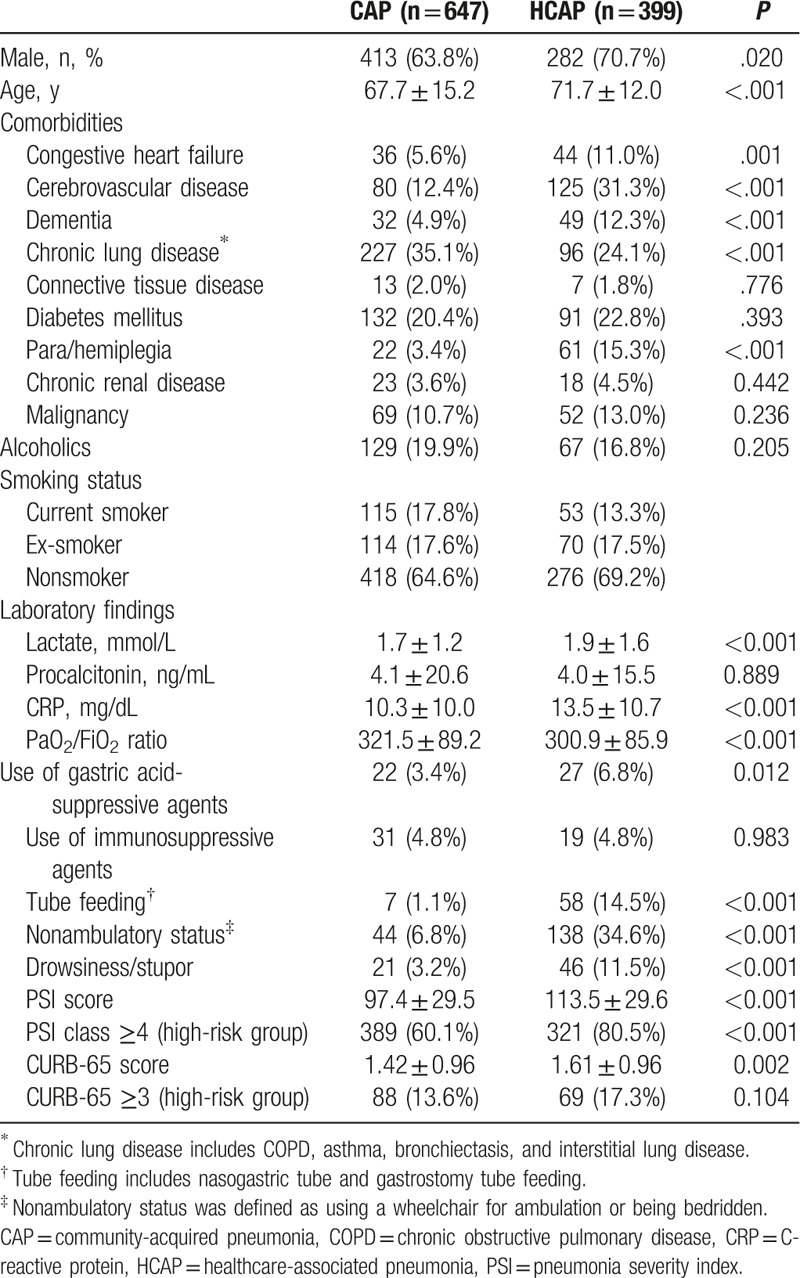
Baseline characteristics of the study patients.

### 3.2 Pathogen distribution

**Figure 1:**
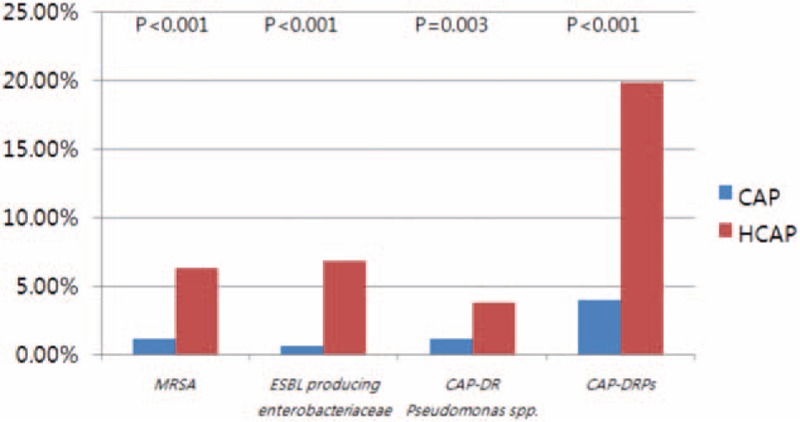
Distribution of CAP-DRPs in patients with HCAP. CAP: community-acquired pneumonia; CAP-DRPs; community-acquired pneumonia drug-resistant pathogens; HCAP: healthcare-associated pneumonia; ESBL: extended-spectrum β-lactamases; MRSA: methicillin-resistant *Staphylococcus aureus*.

Pathogens were identified in 153 (38.3%) of 399 HCAP patients and 177 (27.4%) of 647 CAP patients. The pathogen distribution according to the type of pneumonia is shown in Table 3. In patients with HCAP, *Klebsiella pneumoniae* (8.3%) was isolated most frequently, followed by *Pseudomonas aeruginosa* (7.5%), *Streptococcus pneumoniae* (6.5%), and MRSA (6.3%). In patients with CAP, *S. pneumoniae* (9.0%), *K. pneumoniae* (5.4%), *Mycoplasma pneumoniae* (4.0%), and *Pseudomonas aeruginosa* (4.0%) were the 4 most frequently isolated pathogens. The prevalence of CAP-DRPs in the HCAP group was significantly higher than that in the CAP group (19.8% vs 4.0%, *P* < .001) (Fig. 1). In particular, MRSA and extended-spectrum β-lactamase (ESBL)-producing Enterobacteriaceae species were isolated more frequently in patients with HCAP than in those with CAP (6.3% vs 1.1%, *P* < .001; and 6.8% vs 0.6%, *P* < .001).

**Table 3:**
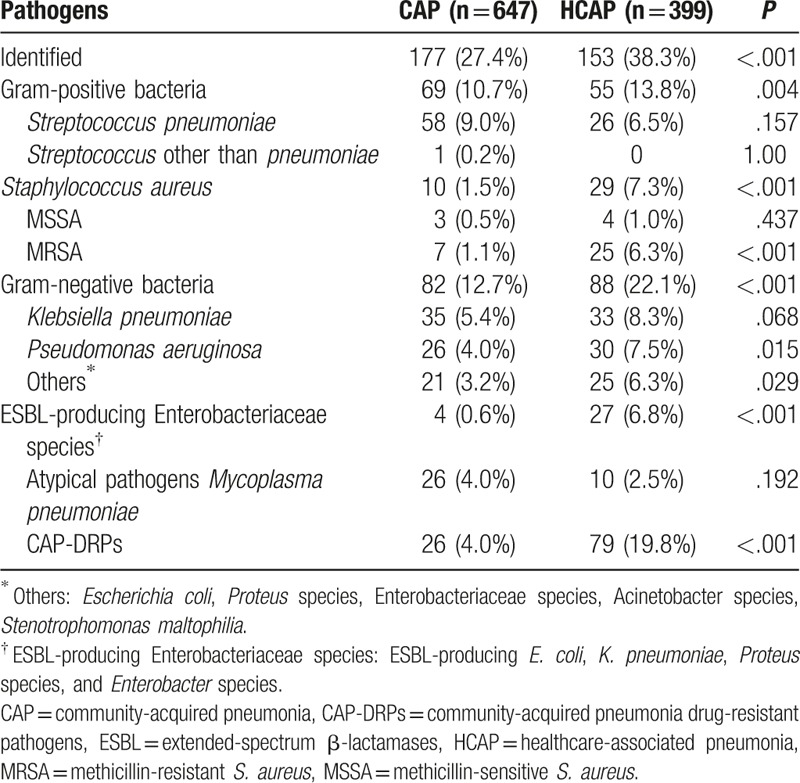
Pathogens identified in patients with CAP and HCAP.

The pathogens that were not appropriately targeted by the initial antibiotic therapy are shown in Table 4. In patient with HCAP, MRSA (29.9%), ESBL-producing *K. pneumoniae* (26.9%), and *P. aeruginosa* (20.9%) were the 3 common pathogens. In patients with CAP, *P. aeruginosa* (30.0%) was the most common pathogen, followed by MRSA (23.3%), and *M. pneumoniae* (23.3%).

**Table 4:**
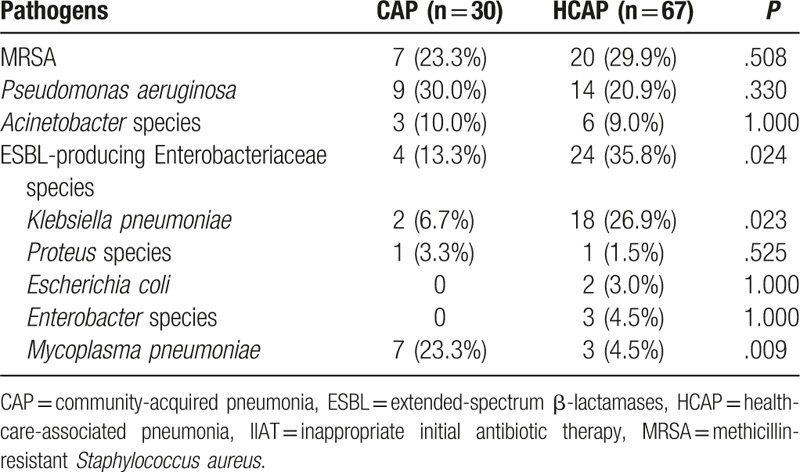
Pathogens targeted by IIAT in patients with CAP and HCAP.

### 3.3 Antibiotic therapy

Initial antibiotic treatments for patients with HCAP and CAP are shown in Table 5. β-lactam with macrolide was the most frequently used antibiotic combination in CAP patients (37.9%). Antipseudomonal cephalosporin/penicillin monotherapy was used most frequently in HCAP patients (47.9%). Antipseudomonal antibiotics were given to 85.7% of patients with HCAP and 56.4% of patients with CAP as initial empirical therapy, although *Pseudomonas* was detected in 7.5% and 4.0% of patients with HCAP and CAP, respectively. Twenty-two (5.5%) and 6 (0.9%) patients with HCAP and CAP received anti-MRSA antibiotics, respectively.

**Table 5:**
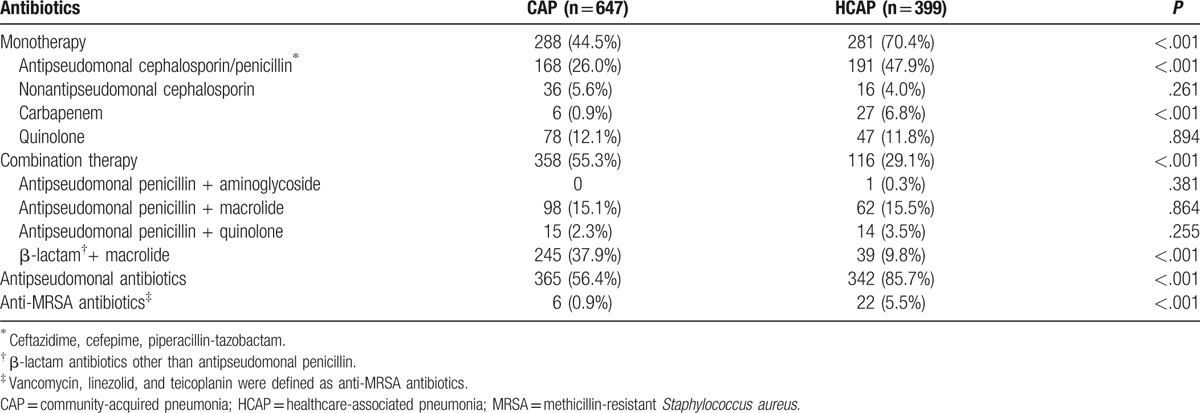
Initial prescribed antibiotics in patients with CAP and HCAP.

### 3.4 Clinical outcomes

CAP-DRPs were isolated more frequently in patients with HCAP than in patients with CAP (19.8% vs 4.0%, *P* < .001) (Table 6). IIAT was administered more frequently to HCAP patients than to CAP patients (16.8% vs 4.6%, *P* < .001). HCAP was associated with not only an increased rate of ICU admission (7.8% vs 4.2%, *P* = .018) but also an increased 28-day mortality rate (14.5% vs 6.3%, *P* < .001). The inhospital mortality rate was also higher in HCAP patients than in CAP patients (13.5% vs 6.6%, *P* < .001). Patients with HCAP stayed in hospital for longer than did patients with CAP (16.1 ± 19.4 vs 10.5 ± 9.6, *P* < .001).

**Table 6:**
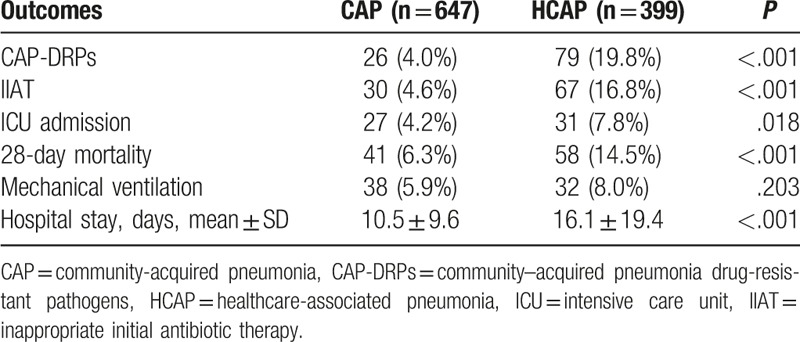
Clinical outcomes according to type of pneumonia.

To investigate the risk factors for 28-day mortality in community-onset pneumonia patients, a multivariable logistic analysis was conducted. In this analysis, mechanical ventilation (OR 2.33, 95% CI 1.25–4.36, *P* = .008), PSI (OR 1.02, 95% CI 1.01–1.03, *P* < .001) and HCAP (OR 1.84, 95% CI 1.18–2.86, *P* = .007) were significant independent risk factors for 28-day mortality in community-onset pneumonia patients (Table 7).

**Table 7:**
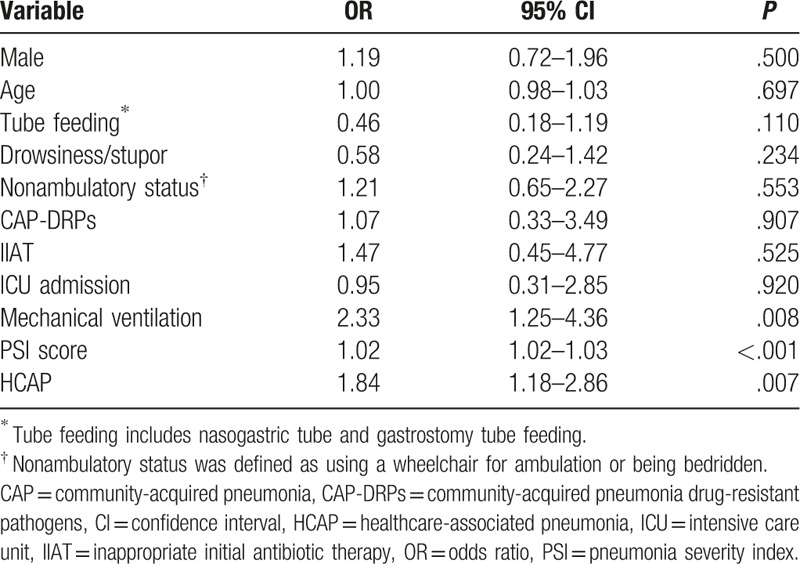
Multivariable analysis of predictors of 28-day mortality in community-onset pneumonia patients (CAP and HCAP combined).

To investigate the risk factors for 28-day mortality in HCAP patients, a multivariable logistic analysis was conducted. In this multivariable analysis, PSI (OR 1.02, 95% CI 1.01–1.04, *P* < .001) was a significant independent risk factor for 28-day mortality in HCAP patients. CAP-DRPs and IIAT were not associated with 28-day mortality in HCAP patients (Table 8). When stratified by PSI score, patients of PSI class ≥ 4 showed a higher mortality rate (OR 5.42, *P* = .031).

**Table 8:**
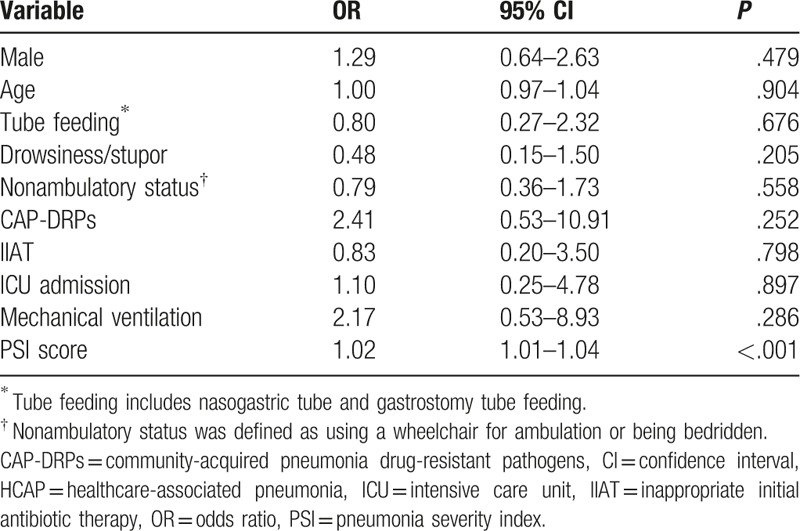
Multivariable analysis of predictors of 28-day mortality in HCAP patients.

### 3.5 Risk factors for the occurrence of CAP-DRPs

Assessment of risk factors was performed by combining data for patients with HCAP and those with CAP. The univariable and multivariable analysis of risk factors for the occurrence of CAP-DRPs in community-onset pneumonia, including HCAP and CAP, is shown in Table 9 and Table 10. The independent risk factors for CAP-DRPs were as follows: tube feeding (OR 4.20; 95% CI, 2.16–8.16), nonambulatory status (OR 2.77; 95% CI, 1.60–4.80), and HCAP (OR 3.16; 95% CI 1.89–5.27).

**Table 9:**
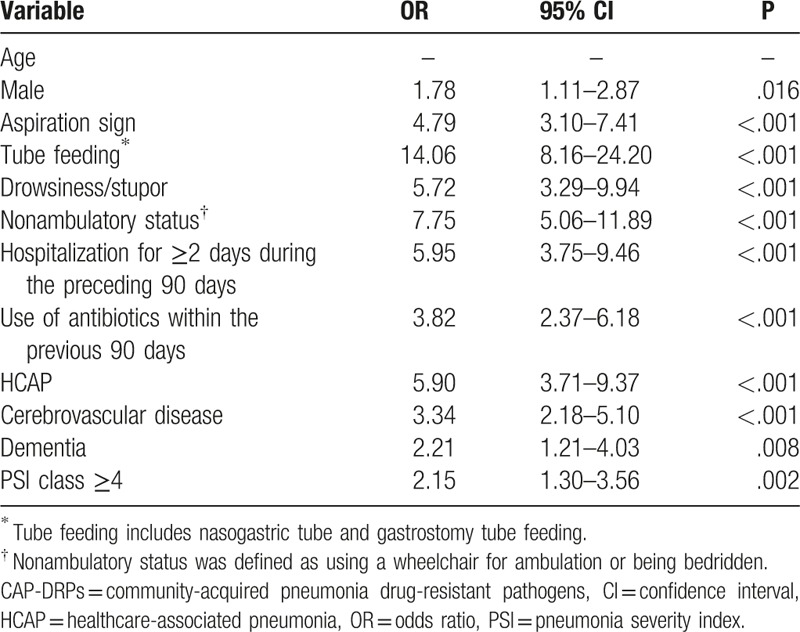
Univariable analysis of risk factors for CAP-DRPs in patients with community-onset pneumonia (CAP and HCAP patients).

**Table 10:**
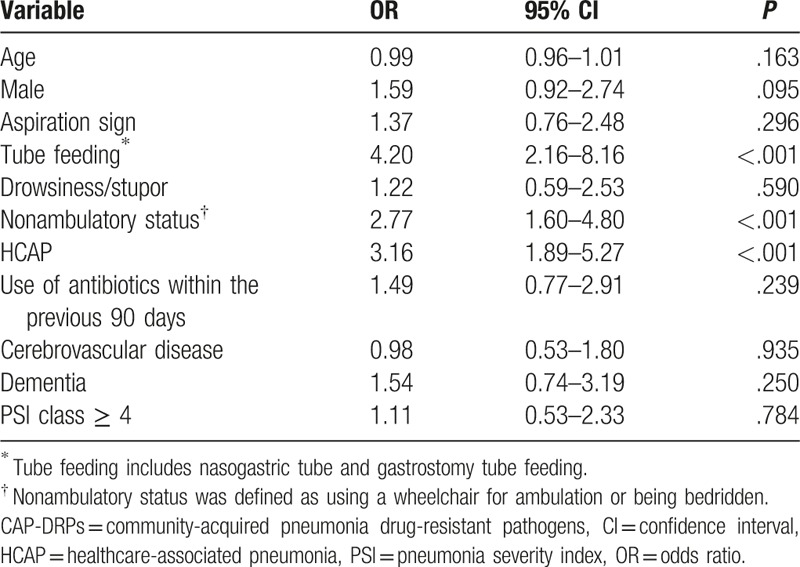
Multivariable analysis of risk factors for CAP-DRPs in patients with community-onset pneumonia (CAP and HCAP patients).

## 4 Discussion

This study revealed that about 38.1% of the hospitalized pneumonia patients in a tertiary teaching hospital in South Korea were classified as HCAP. Patients with HCAP were older and more likely to have comorbidities than patients with CAP. PSI, presence of CAP-DRPs, and administration of IIAT were also more frequent in patients with HCAP than in those with CAP. However, in a multivariable analysis, PSI rather than the presence of CAP-DRPs or IIAT was an independent factor predictive of mortality in patients with HCAP. Independent risk factors for the occurrence of CAP-DRPs were the presence of tube feeding, nonambulatory status, and HCAP itself in all pneumonia patients.

To our knowledge, this is the largest study to compare the epidemiology and outcomes of HCAP and CAP patients in South Korea. The incidence of HCAP in our study was similar to those in previous reports from other countries that have reported the incidence among hospitalized patients (32.5%–49.5%).^[5,10,11]^ Several studies in South Korea have reported higher incidences (43.0%–52.8%) of HCAP than that in our study (38.1%).^[12–14]^

In our study, the rate of CAP-DRPs in HCAP patients was significantly higher than that in CAP patients, as in other studies.^[5,12–14]^ ESBL-producing Enterobacteriaceae, including ESBL-producing *K. pneumoniae*, were the most common CAP-DRPs in patients with HCAP. The incidence of MRSA and CAP drug-resistant *P. aeruginosa* in HCAP patients was significantly higher than that in CAP patients. In addition, the proportions of initial administration of antipseudomonal and anti-MRSA antibiotics in the HCAP patients were higher than those in CAP patients according to the 2005 ATS/IDSA guidelines.^[1]^ Despite the more frequent use of anti-pseudomonal (85.7%) and anti-MRSA (5.5%) antibiotics in the HCAP patients, IIAT was more frequent in patients with HCAP (16.8%) than in those with CAP (4.6%).

In our study, the clinical course was worse and the duration of hospital stay was longer in HCAP patients than in CAP patients, as previously reported.^[13,15–17]^ In a logistic regression analysis, PSI was a significant independent risk factor for 28-day mortality in HCAP patients. Presence of CAP-DRPs and IIAT were not associated with mortality in HCAP patients. This is possibly because not CAP-DRPs and IIAT but disease severity itself is related to mortality.

According to the 2005 ATS/IDSA guidelines, the fundamental concept of HCAP was that the presence of CAP-DRPs suggests more IIAT, resulting in high mortality. Hence, initial broad-spectrum antibiotics were recommended to improve clinical outcomes.^[1]^ However, this concept of HCAP has come into question recently. Several studies reported that patients with HCAP are not at high risk for drug-resistant pathogens.^[2,10,18]^ The occurrence of drug-resistant pathogens is associated not only with contact with healthcare systems but also underlying patient characteristics.^[2,6,10,19]^ Moreover, increased mortality in HCAP patients was also shown to be related to underlying patient-related factors and the severity of illness, rather than the presence of CAP-DRPs or IIAT.^[11,19,20]^ A cohort study conducted at 346 United States hospitals found no evidence that guideline-based antimicrobial therapy reduced mortality in HCAP patients.^[21]^ It is increasingly recognized that comorbidities account for a large proportion of mortality in patients with HCAP and a significant proportion of this mortality cannot be prevented by antibiotic treatment.^[2]^ In this context, the newest 2016 ATS/IDSA guidelines removed the concept of HCAP. Our study also showed that high mortality in HCAP patients is associated with underlying patient-related factors and the severity of the illness, rather than with the presence of CAP-DRPs or IIAT in Korea. Therefore, we consider that the concept of HCAP could be removed in the management of pneumonia patients in Korea.

However, it is still important to detect those patients at high risk of drug-resistant pathogens, and to manage them using early appropriate antibiotic coverage. Our study showed that tube feeding, a nonambulatory status, and HCAP are risk factors for the occurrence of CAP-DRPs in community-onset pneumonia patients. Therefore, care is needed when selecting initial empirical antibiotics in such patients. Based on our study, patients with tube feeding, a nonambulatory status, and HCAP should be considered to start initial antibiotics covering MRSA, ESBL-producing microorganisms, and *P. aeruginosa.* Further studies are required to clarify the risk factors for the occurrence of CAP-DRPs in community-onset pneumonia patients.

Here, we identified three independent risk factors for CAP-DRPs in community-onset pneumonia. In this study, 2 risk factors not included in the HCAP definition were identified. Poor functional status (tube feeding, nonambulatory status) was also an independent risk factor for CAP-DRPs. Patients with poor functional status are prone to cross-contamination of bacteria due to an increased requirement for care.^[22]^ Shindo et al^[5]^ also reported that poor functional status (tube feeding, nonambulatory status) is a risk factor for CAP-DRPs. Overall, these results suggest that functional impairment is the most significant factor for the occurrence of CAP-DRPs.

There were several limitations to our study. First, it was a retrospective study at a single center in South Korea, which had a large number of cerebrovascular disease and chronic lung disease patients. Thus, the results cannot be generalized. The medical records regarding restrictions of treatment escalation, such as do-not-resuscitate orders, were insufficient. Therefore, the influence of restrictions of treatment escalation on mortality was not considered. Second, the rate of pathogen identification was low. Thus, the incidence of CAP-DRPs could have been underestimated. However, our pathogen identification rate (38.3%) was higher than those reported previously.^[13,20]^ Third, the microbiologic data were obtained mainly from sputum cultures, and were semiquantitative. Hence, their accuracy is uncertain. The evaluation of atypical pathogens such as *Chlamydia, Legionella*, and viruses was also insufficient because of incomplete medical records. Fourth, as we enrolled only pneumonia patients who required hospitalization, our results are not applicable to outpatients.

In conclusion, HCAP was associated with increased rates of CAP-DRPs, IIAT, and 28-day mortality. However, the increased mortality in HCAP patients was related to underlying patient-related factors and the severity of illness assessed by PSI rather than the presence of CAP-DRPs or IIAT. Thus, future antibiotic strategies for pneumonia patients should be based on PSI and established risk factors for CAP-DRPs, not merely on contact with the healthcare system. Therefore, we consider that the 2016 ATS/IDSA guidelines could be utilized in real practice in Korea.
