# Clinical characteristics and prognostic risk factors of healthcare-associated pneumonia in a Korean tertiary teaching hospital: Erratum

**DOI:** 10.1097/MD.0000000000009319

**Published:** 2017-12-15

In the article, “Clinical characteristics and prognostic risk factors of healthcare-associated pneumonia in a Korean tertiary teaching hospital”,^[1]^ which appeared in Volume 96, Issue 42 of *Medicine*, three of the authors’ names should appear as June Hong Ahn, Jin Hong Chung and Choong Ki Lee.
